# The Severity of Experimental Autoimmune Cystitis Can be Ameliorated by Anti-CXCL10 Ab Treatment

**DOI:** 10.1371/journal.pone.0079751

**Published:** 2013-11-21

**Authors:** Udai P. Singh, Narendra P. Singh, Honbing Guan, Venkatesh L. Hegde, Robert L. Price, Dennis D. Taub, Manoj K. Mishra, Mitzi Nagarkatti, Prakash S. Nagarkatti

**Affiliations:** 1 Pathology, Microbiology and Immunology, School of Medicine, University of South Carolina, Columbia, South Carolina, United States of America; 2 Department of Cell and Developmental Biology, University of South Carolina, Columbia, South Carolina, United States of America; 3 Hematology and Immunology Research, VA Medical Center, Department of Veteran Affairs, Washington DC, United States of America; 4 Department of Math and Sciences, Alabama State University, Montgomery, Alabama, United States of America; Wayne State University, United States of America

## Abstract

**Background:**

Interstitial cystitis (IC), more recently called painful bladder syndrome (PBS) is a complex disease associated with chronic bladder inflammation that primarily affects women. Its symptoms include frequent urinary urgency accompanied by discomfort or pain in the bladder and lower abdomen. In the United States, eight million people, mostly women, have IC/PBS. New evidence that autoimmune mechanisms are important in the pathogenesis of IC/PBS triggered interest.

**Methodology/Principal Findings:**

SWXJ mice immunized with a homogenate of similar mice’s urinary bladders develop an autoimmune phenotype comparable to clinical IC with functional and histological alterations confined to the urinary bladder. Using the murine model of experimental autoimmune cystitis (EAC), we found that serum levels of CXCR3 ligand and local T helper type 1 (Th1) cytokine are elevated. Also, IFN-γ-inducible protein10 (CXCL10) blockade attenuated overall cystitis severity scores; reversed the development of IC; decreased local production of CXCR3 and its ligands, IFN-γ, and tumor necrosis factor-α (TNF-α); and lowered systemic levels of CXCR3 ligands. Urinary bladder CD4^+^ T cells, mast cells, and neutrophils infiltrates were reduced following anti-CXCL10 antibody (Ab) treatment of mice. Anti-CXCL10 Ab treatment also reversed the upregulated level of CXCR3 ligand mRNA at urinary bladder sites. The decreased number and percentage of systemic CD4^+^ T cells in EAC mice returned to normal after anti-CXCL10 Ab treatment.

**Conclusion/Significance:**

Taken together, our findings provide important new information about the mechanisms underlying EAC pathogenesis, which has symptoms similar to those of IC/PBS. CXCL10 has the potential for use in developing new therapy for IC/PBS.

## Introduction

Interstitial cystitis (IC) is a complex disease resulting from an inflammatory condition of the bladder wall; it is characterized by chronic urinary frequency and urgency accompanied by discomfort or pain in the bladder and lower abdomen. IC primarily affects women. It is estimated that as many as one million people in the United States are affected by IC [1], [2], [3], [4]. The term “painful bladder syndrome (PBS)” has recently been used to describe the disease [5], [6], while “IC” has been applied only to patients who demonstrate the characteristic histological and cystoscopic findings [7]. The etiology and pathogenesis of IC remain unknown. The potential pathophysiologic causes of IC include inflammatory, autoimmune, neurogenic, vascular, and/or lymphatic disorders, all resulting in similar clinical manifestations. It has been shown that, urinary bladder affected by chronic IC infiltrated by T cells, monocytes, mast cells, and plasma cells [8], [9].

In the past 20 years, many animal models have been used to investigate the pathogenesis of IC [10], but such models only partially mimic the human IC phenotype. Since a recent report associated IC with other disease states having an autoimmune etiology, among them systemic lupus erythematosis, rheumatoid arthritis, ulcerative colitis, and thyroiditis [11], the possibility that IC also has autoimmune pathogenesis has engaged the scientific community.

Recently, the use of experimental autoimmunity, achieved by inducing a proinflammatory Type 1 T-cell response (Th1) to a targeted self-antigen, has contributed to the creation of useful models of autoimmune types of encephalomyelitis [12], myocarditis [13], oophoritis [14], and cystitis (EAC) [15]. The EAC model, which mimics the phenotype of human IC, has been well described [15], [16]. EAC mice develop many IC/PBS characteristics, such as increased frequency of urination, decreased bladder capacity, decreased intercontraction interval, decreased urine output per void, urothelial detachment, and increased bladder permeability with epithelial leakage [15]. To the best of our knowledge, this is the only mouse model that mimics human IC/PBS pathogenesis and, most importantly, is mediated by bladder autoimmune responses.

Chemokines have emerged as major factors in inflammatory diseases. CXCR3 and its ligands CXCL9, CXCL10, and CXCL11 are differentially elevated in many instances, such as with periodontal [17], [18], autoimmune liver diseases [19], multiple sclerosis [20], bronchiolitis [21], skin or mucosal inflammation [22], cyclophosphamide (CYP)-induced cystitis [23], and inflammatory bowel disease (IBD) [24], [25]. Interestingly, IBD is common in IC patient populations [26], [27]. Also, as compared to the general population, individuals with IC are 100 times more likely to develop IBD. We have shown that serum levels and mRNA expression of CXCL9, CXCL10, and CXCL11 are increased in human IC, as well as in CYP-induced cystitis in the urinary bladder and iliac lymph nodes (ILN) [23].

The present study demonstrates that modulation of a CXCR3 ligand (CXCL10) interaction ameliorates the disease severity in a recently developed EAC model of IC. The findings from this study will help in the development of improved treatment protocols for IC as well as justify future correlative studies to identify CXCL10 levels as a valid non-invasive marker for IC/PBS like condition.

## Results

### Systemic CXCL9, CXCL10, and CXCL11 Levels Increase in EAC Mice

**Figure 1:**
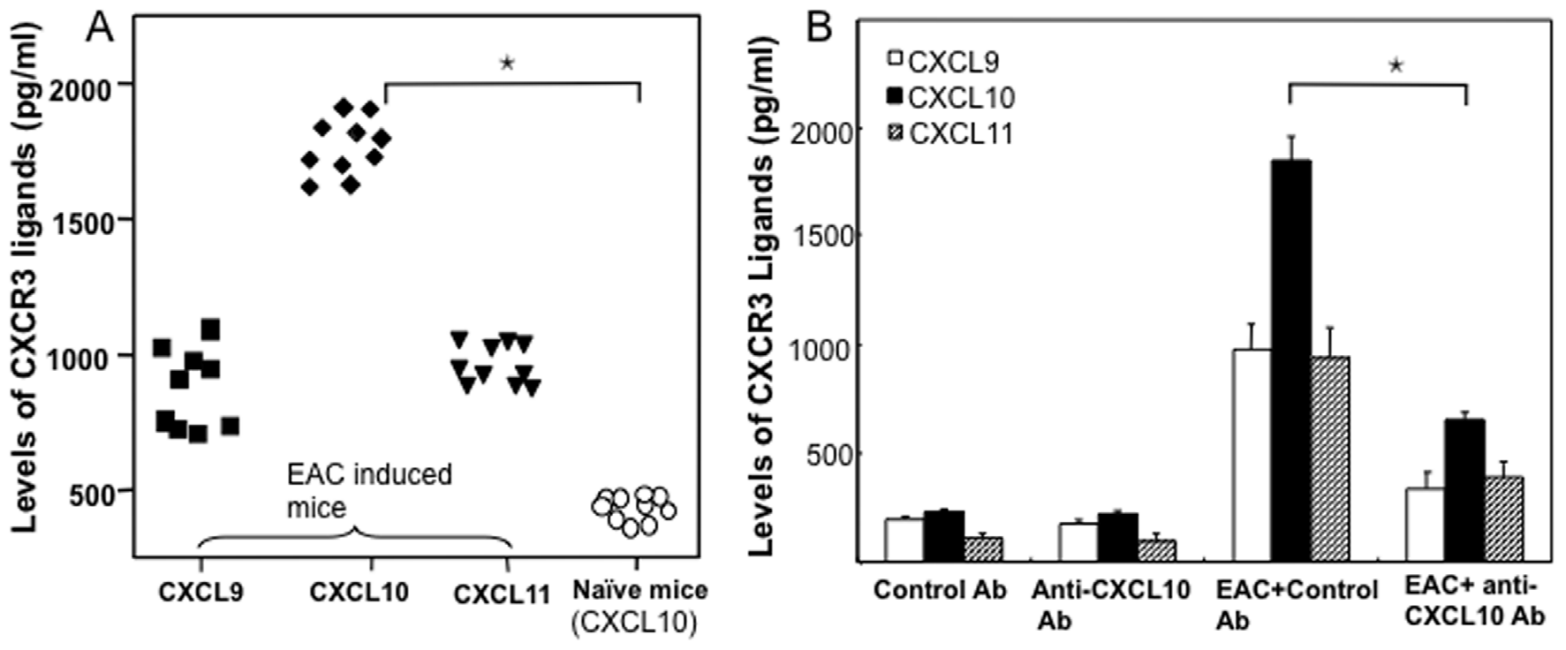
EAC was induced by injecting female SWXJ mice sc with 0.2 ml emulsion and 0.1 ml DDDH_2_O containing lyophilate from SWXJ mice with 0.1 ml of complete Freund’s adjuvant (CFA) containing 400 µg of *Mycobacterium tuberculosis* H37R1. Changes in systemic CXCL9, CXCL10 and CXCL11 levels in EAC mice and CXCL10 in naive mice were measured 3 month after EAC induction (panel A). After 3 months of EAC induction, control or anti-CXCL10 Ab solutions were administered every 2 days for eight weeks. At the end of experiments, serum levels of CXCL9 (▪), CXCL10 (♦), CXCL11 (▾) in experimental and naïve mice (○) (panel B) were determined by ELISA assays. The data presented are the mean concentrations in each group. (A) Asterisks indicate statistically significant differences, i.e., *p*<0.01 (*), between naïve and CXCL10 groups. (B) *p*<0.01 (*), between CXCL10 level of EAC-induced control and anti-CXCL10 Ab-treated groups.

In a previous study, we demonstrated that serum levels of CXCL9, CXCL10, and CXCL11 were significantly higher in IC patients than in normal donors [23]. Further, others and we have shown that these CXCR3 ligands mainly attract activated T cells of the Th1 phenotype, which express high levels of CXCR3 [25], [28]. Our previous clinical findings correlate with results from this study using the EAC model, which showed a similar increase in serum CXCR3 ligand levels and correlated well with the severity of cystitis disease as compared to that in control mice (Fig. 1A). EAC mice expressed higher serum CXCL10> CXCL9> CXCL11 levels than did naïve mice (Fig. 1A).

Because of the high levels of CXCL10 observed during EAC, we next treated these mice with control or anti-CXCL10 Ab to determine whether inhibition of this chemokine ligand would modulate the severity of cystitis. Unaffected mice treated with either control or anti-CXCL10 Ab showed no significant change in serum CXCR3 ligand levels (Fig. 1B). However, the increases in serum CXCL10 concentrations were significantly reduced after CXCL10 blockade (Fig. 1B). We also noted a moderate reduction in CXCL9 and CXCL11 levels after CXCL10 blockade.

### CXCL10 Blockade Reduces Cystitis Severity

**Figure 2:**
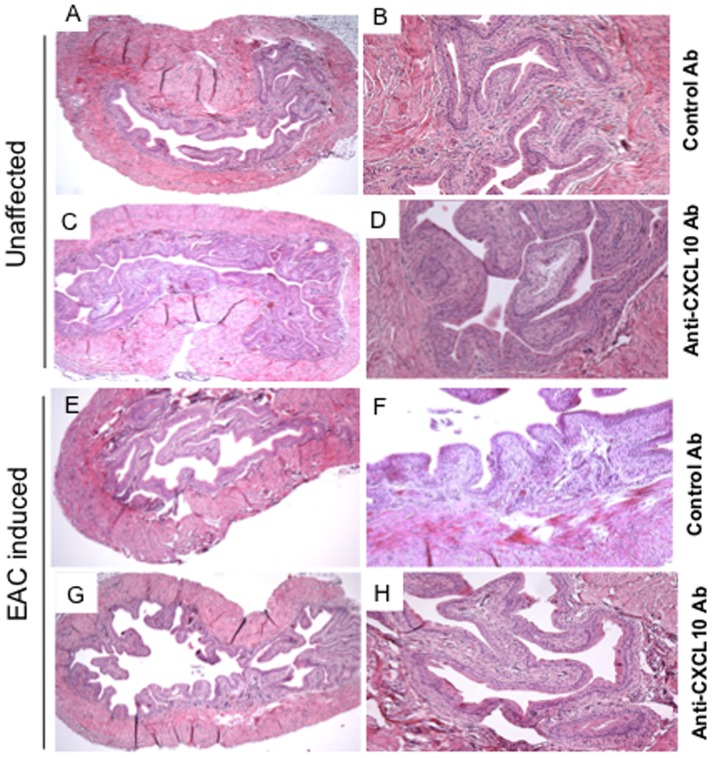
Histopathological changes in the urinary bladders of chronic EAC mice treated with anti-CXCL10 Ab. Bladder sections from control or anti-CXCL10 Ab groups are shown at different magnifications (A, C, E, G X30; B, D, F, H X100). Panel F shows sections from EAC-induced mice treated with control Ab to illustrate inflamed bladders characterized by differences in mucosal wall thickness, enlargement of the mucosal layer, and leukocyte infiltration. Panel H shows marked improvement after anti-CXCL10 Ab treatment.

Despite the reduction in CXCL10 in mice following EAC and anti-CXCL10 Ab treatment, it remains to be determined whether CXCL10 blockade will reverse EAC severity. In normal mice, the stroma of the urinary bladder has attached, well-built connective tissue with few leukocyte infiltrates (Fig. 2A). EAC mice showed pathological findings similar to those of IC in the urinary bladder mucosa (Fig. 2). The urinary bladder lining of control Ab-treated mice was much larger than the bladder lining of similar EAC mice treated with anti-CXCL10 Ab (Fig. 2F). A reduction in the severity of EAC was manifested as a decrease in urinary bladder mass and the presence of fewer leukocyte infiltrates, as well as a lower disease score in the anti-CXCL10 Ab- treated group than in control Ab-treated mice (Fig. 2G, H; Table 1). The urinary bladder stroma of these mice consists of loose connective tissue with intense, mixed leukocyte infiltrates mainly composed of polymorphonuclear cells and lymphocytes, as shown by flow cytometry analysis. We provide the first demonstration that anti-CXCL10 Ab treatment reverses the onset of EAC in mice. Most importantly, inhibition of the critical CXCR3 ligand reduces the expression of CXCL10, CXCL11, and CXCL9, thus limiting CXCR3-positive cells to sites of inflammation in the urinary bladder of EAC mice.

**Table 1:** Histological evaluations of mice with or without EAC-induced cystitis after control and/or anti-CXCL10 Abs treatment.

Treatment Group	Number of Mice	Cystitis Disease Score (0–4)
Control Abs treated	15	0
Anti-CXCL10 Abs treated	15	0
EAC-induced+ control Abs treated	15	3.43±0.52
EAC-induced+ anti-CXCL10 Abs treated	15	1.21±0.92?

At the end of experiment the severity of urinary bladder inflammation was evaluated by histopathological changes described in the methods section for EAC induction and treatment. Mice were sacrificed at the experimental end point and urinary bladders was fixed in 10% neutral formalin for 24 hrs and embedded in paraffin. Fixed tissues were sectioned at 6 µm, stained with hematoxylin and eosin, and examined by light microscopy. The inflammatory state of each urinary bladder was characterized and scored as described in the methods section. The studies were repeated 3 times, and the data presented are the mean score changes ± SEM of these experiments. Differences between anti-CXCL10 Abs treated and EAC induced control Abs treated group were considered significant when *p*<0.01 (*ζ*).

### Reduction in the Expression of Mucosal CXCL9, CXCL10, CXCL11, IFN-γ, IL-12p40, and TNF-α Following Anti-CXCL10 Ab Treatment

**Figure 3:**
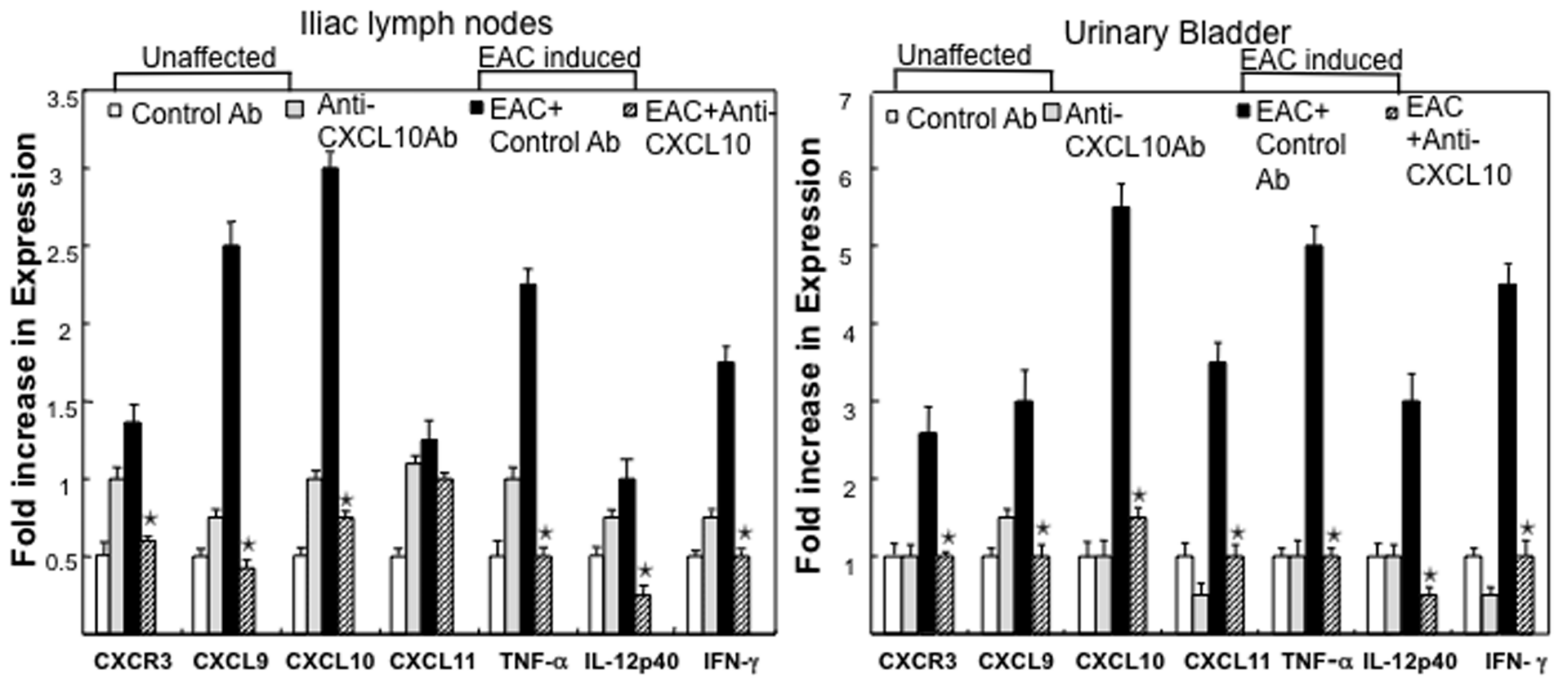
Changes in CXCR3, CXCL9, CXCL10, CXCL11, TNF-α, IL-12p40 and IFN-γ mRNA expressions by EAC mice after anti-CXCL10 Ab treatment. At the experimental end point, total RNA was isolated from the immune cells of iliac lymph nodes, or urinary bladder of the mice.RT-PCR analysis of unaffected group control Ab [white bar], Anti-CXCL10 Ab [gray bar], EAC induced group EAC+ control Ab [black bar], or EAC+ anti-CXCL10 Ab [striped bar] mRNA expression was performed. Fold increase in expression were expressed. Asterisk(s) (*) indicate statistically significant (*p*<0.05) increases between the EAC+ control Ab-treated group and the EAC group that received anti-CXCL10 Ab.

We investigated local changes in the expression of Th1 and inflammatory cytokines and CXCR3 ligands at the peak of chronic EAC. The levels of CXCL9, CXCL10, CXCL11, IFN-γ, IL-12p40, and TNF-α mRNAs were measured by quantitative RT-PCR analysis. EAC mice receiving the control Ab exhibited a substantial increase in the expression of CXCR3 and its ligands, IFN-γ, IL-12p40, and TNF-α mRNA, in the immune cells of ILN and urinary bladders when compared with control Ab-treated unaffected mice (Fig. 3). The expression of IFN-γ, IL-12p40, and TNF-α mRNA by immune cells in the ILN and urinary bladders of EAC-induced mice was significantly decreased following anti-CXCL10 Ab treatment as compared with similarly affected mice treated with control Ab. Hence, the results suggest that CXCL10 blockade significantly inhibited the production of these cytokines and reduced inflammation at the ILN (inductive site) and urinary bladder (effector site).

### Modulation of CD4^+^ Lymphocytes by Anti-CXCL10 Ab Treatment after EAC Induction

**Figure 4:**
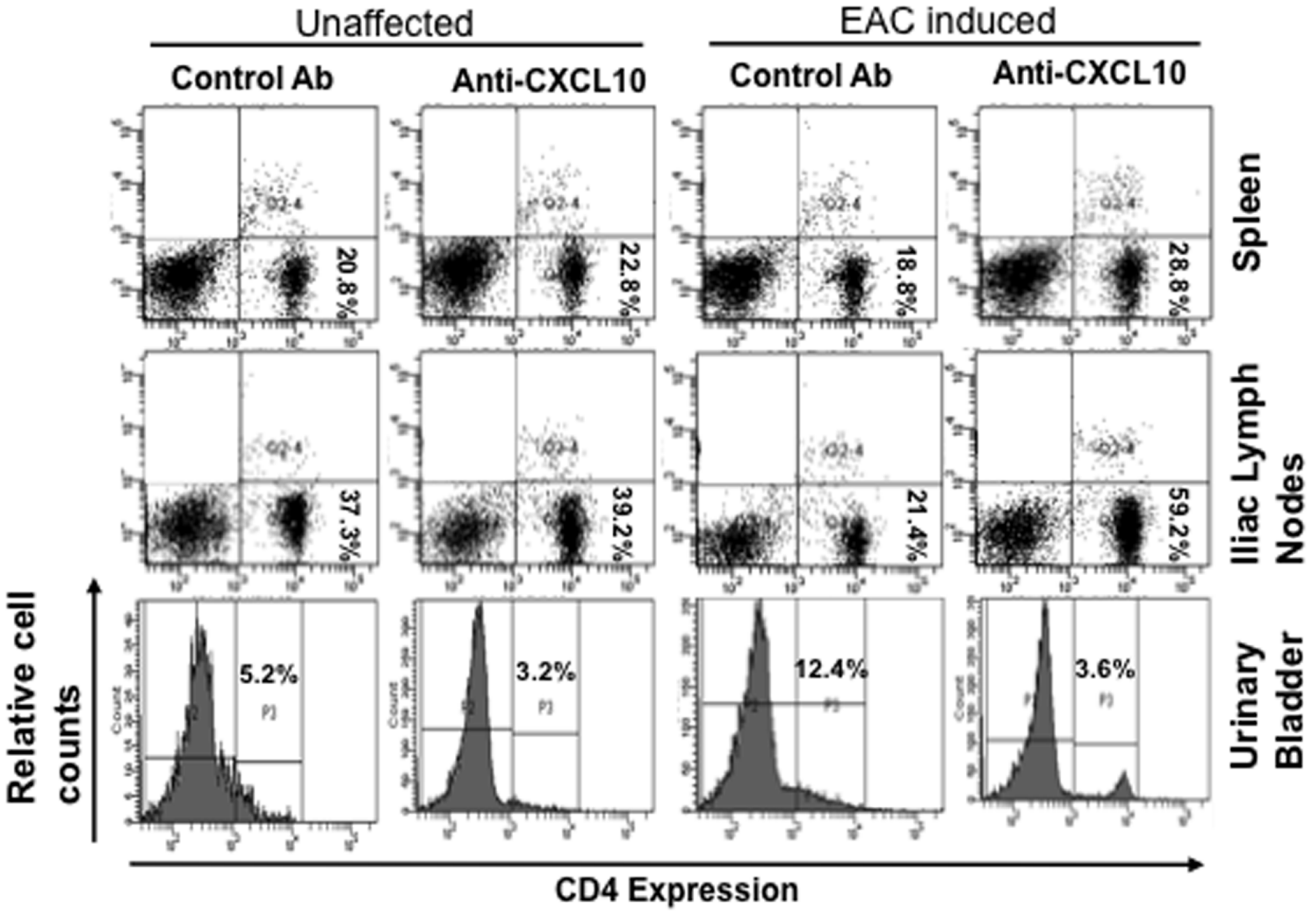
Change in number of CD4^+^T-cell lymphocytes in EAC mice after anti-CXCL10 Ab treatment. At the end of experiments, lymphocytes from the spleen, iliac lymph nodes, and urinary bladder were isolated, stained for CD4^+^ T cell expression, and analyzed by flow cytometry. A representative histogram is shown with the mean percentage of spleen, iliac lymph node, and urinary bladder CD4^+^ cells per mouse. The data presented are representative dot plots and histograms from three independent experiments involving 5 mice per group.

To demonstrate the autoimmune response in EAC mice, we used flow cytometry analysis to determine the changes in populations of T cells (CD4^+^) in the spleens, ILNs, and urinary bladders of EAC mice treated with control and/or anti-CXCL10 Ab. The frequency of CD4^+^ lymphocytes increased significantly in the urinary bladder during the disease severity of EAC. We did not note any change in CD4^+^ T cells in the urinary bladders, ILNs, and spleens of unaffected control mice and/or mice treated with anti-CXCL10 Ab alone (Fig. 4). After the induction of EAC, the percentage of CD4^+^ T cells slightly decreased compared to the total leukocyte population in the spleen, but was drastically reduced in ILN. However, anti-CXCL10 Ab treatment increased the percentage of CD4^+^ T cells in the spleen and ILN back to normal, as in unaffected mice. Moreover, significantly increased CD4^+^ T cells in the urinary bladder after EAC induction are reduced after anti-CXCL10 Ab treatment. Correspondingly, the total number of CD4^+^ T cells from the spleens of EAC mice decreased following CXCL10 blockade (data not shown). These findings indicate that the induction of EAC considerably reduced the percentage of T helper lymphocytes in the spleen and iliac lymph nodes, but increased the frequency of CD4^+^ T cells in the urinary bladder. However, CXCL10 blockade increased CD4^+^ T lymphocytes in the spleen, ILN and decreased them in the urinary bladder. This might arbitrate the severity of IC.

### CXCL10 Ab Treatment Reduces the Frequency of Mast Cells after Chronic EAC

**Figure 5:**
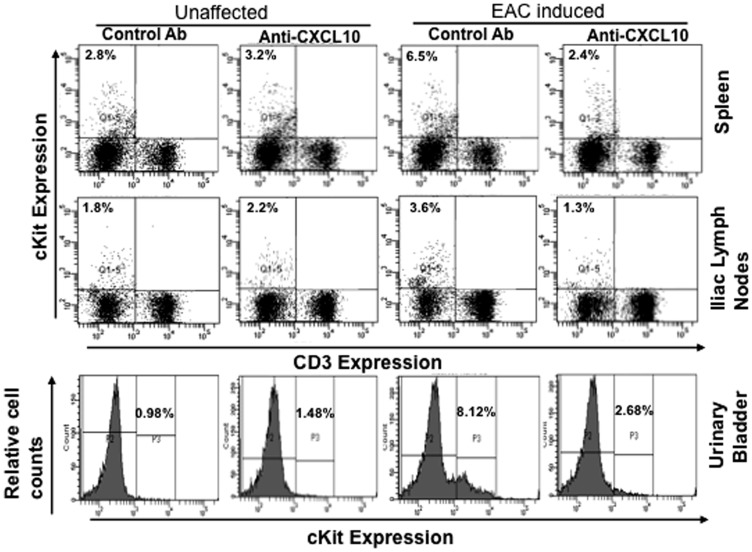
Change in number of mast cells in EAC mice after anti-CXCL10 Ab treatment. At the end of experiments, cells from the spleen, iliac lymph nodes, and urinary bladders were isolated, stained for CD3^+^ and CD117^+^cell (cKit- mast cells) expression, and analyzed (CD3^-^ CD117^+^) by flow cytometry. Histograms show mean percentage of cKit expression. The data presented are representative dot plots and histograms from three independent experiments involving 5 mice per group.

To better define the role of mast cells in the progression of EAC, we next examined changes in mast cell numbers after EAC induction in mice. We assessed changes in the mast cell populations in spleens, ILNs, and urinary bladders after the induction of EAC in mice treated with anti-CXCL10 or control Ab. Normal mice treated with control or anti-CXCL10 Ab showed no changes in the percentages of mast cells in the spleen, urinary bladder, or ILN (Fig. 5). However, mast cells were increased in the spleens, ILNs, and urinary bladders of mice with induced EAC (Fig. 5). These increases were significantly reduced, mainly in the ILN and urinary bladder, after anti-CXCL10 Ab treatment. IC induction considerably increased the percentage of mast cells in the spleen, ILN and urinary bladder, while CXCL10 blockade substantially decreased the percentage and number of mast cells in these organs.

### Anti-CXCL10 Modulates Neutrophils after EAC Induction

**Figure 6:**
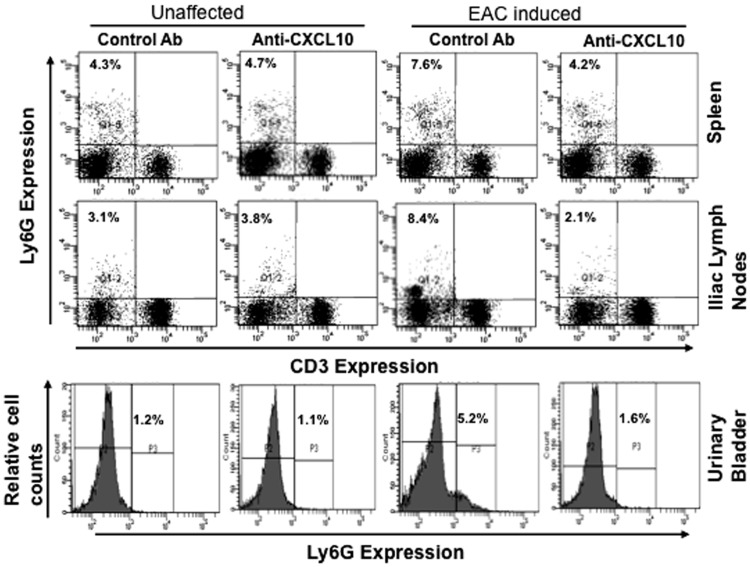
Control or anti-mouse CXCL10 Ab solutions were administered 3 months after EAC induction and every 2 days thereafter for eight weeks. At the end of experiments, cells from the spleens, iliac lymph nodes, and urinary bladders were isolated and stained for Ly6G (neutrophils); expression was analyzed by flow cytometry. Histogram shows mean percentage in LY6G (neutrophils). The data presented are representative dot plots and histograms from three independent experiments involving 5 mice per group.

Neutrophils have the capacity to release CXCL10 [29] and increase the concentration of neutrophil elastase in the urine of cystitis patients [30]. Therefore, to better determine the role of neutrophils in EAC progression, we examined the changes in neutrophils after EAC induction in mice, finding that neutrophils were significantly increased in the spleen, ILN, and urinary bladder after the induction of EAC as compared to control animals (Fig. 6). These populations were significantly reduced after anti-CXCL10 Ab treatment. Taken together, these findings show that during EAC severity there is a considerable increase in the percentage of neutrophils in spleen, ILN, and urinary bladder, but that CXCL10 blockade substantially decreases the percentages of neutrophils in these sites (Fig. 6). In contrast, no significant changes occurred in the percentage of neutrophils as compared to those in unaffected mice given similar treatment with anti-CXCL10 Ab alone.

## Discussion

The etiology and pathogenesis of IC remain unknown, but several pathophysiological mechanisms are implicated in the development of IC/PBS. Although research efforts have focused on elucidating the interrelated mechanisms responsible for this disease, advances have been hampered by the lack of animal models that mimic the IC/PBS phenotype. Increasingly, however, reports have linked IC/PBS with the autoimmune etiology that is responsible for lupus erythematosis, rheumatoid arthritis, ulcerative colitis, and thyroiditis [11].

We have shown that serum levels of CXCR3 ligands are elevated in IC patients as compared to normal donors and in mice with CYP-induced cystitis [23]. We have also shown that anti-CXCL10 antibody (Ab) treatment abrogates the development of CYP-induced cystitis in mice [23]. In the present study, we demonstrated that elevated systemic and mucosal CXCR3 ligand levels correlate with chronic EAC. This murine model displays an autoimmune phenotype and shows functional, histological alterations confined to the urinary bladder. The EAC model also mimics many of the clinical (urinary frequency and decreased urine output per void) and histopathological features of human IC/PBS, as well as a significant decrease in bladder urothelial detachment, increased bladder permeability with epithelial leakage, lymphocyte infiltration, fibrosis, and edema [15], [16]. The EAC mice share some of the features with clinical IC/painful bladder syndrome and show promise for a future transitional outcome.

In this study, using the EAC model, we demonstrated that blocking of CXCR3 ligands with anti-CXCL10 Ab reduced mucosal and systemic proinflammatory cytokines, as well as infiltrated T cells, mast cells and neutrophils, thereby reducing the severity of chronic IC.

We also found that the histopathology of EAC mice clearly shows an increase in thickness of the urinary bladder lamina propria, as well as infiltration of lymphocytes and mast cells in the mucosa and submucosa of the bladder. Taken together, due to similarity with human IC, EAC mice can be used for therapeutic protocols. In the future, CXCR3 ligands levels can be developed as noninvasive prognostic markers for chronic IC severity.

The data at our disposal clearly suggest the direct involvement of immune-cell-mediated production of cytokines/chemokines in the pathogenesis of EAC in mice. The present study shows that bladders’ immune cells are characterized by an increase in proinflammatory Th1 response. IFN-γ is an important cytokine that Th1 cells initially produce in autoimmune responses [31]. Further, IFN-γ can induce CXCR3 and its ligand response [32], [33]. TNF-α is produced by Th1 cells and macrophages, and is involved at sites of inflammation [34]. The role of TNF-α in cystitis has been well reported [35], [36], [37], [38]. An increase in the level of TNF-α has been observed in the urine of IC patients [39]. The other cytokine, IL-12, also activates STAT1, but this mechanism is mediated by increased serine (not tyrosine) phosphorylation. It remains uncertain whether IL-12 alone can enhance STAT1 activation for subsequent CXCR3 ligand expression [40], but this important Th1 differentiator is expressed in the urinary bladder during EAC progression. In the present study, IFN-γ, TNF-α, IL-12p40, and CXCR3, as well as its ligand transcripts, were significantly increased in immune cells of the ILN and urinary bladder in EAC mice. Thus, modulation of CXCR3 ligands in the urinary bladder and ILN can be explained by the presence of various pro-inflammatory cytokines. These inflammatory cytokines were also affected by CXCL10 blockade after EAC severity. We found that IFN-γ, TNF-α, IL-12p40, CXCR3, and its ligand mRNA levels were significantly reduced after anti-CXCL10 Ab treatment, suggesting that these chemokines/cytokines contribute to the exacerbation of EAC.

CXCR3 ligands (CXCL9, -10, and -11) have 40% amino acid sequence identity, secreted by endothelium, epithelium, fibroblasts, keratinocytes, neutrophils, monocytes, and dendritic cells. These ligands are chemotactic for leukocytes and activated T lymphocytes that express CXCR3 [32], [41]. CXCR3 ligands, with an order of efficiency of CXCL11> CXCL10> CXCL9 differentially induce chemotaxis of inflammatory Th1 cells [42]. In this study, we observed a significant increase in the level of CXCL10 as compared to CXCL9 and CXCL11. Due to sequence identity in these CXCR3 ligands and mixed expression by various immune cell types, blocking CXCL10 might affect other ligands at cellular levels, resulting in modest systemic reduction of CXCL9 and CXCL11, as seen in this study.

The autoimmunity theory of the pathophysiology of IC has triggered interest in recent years as a result of reports of association between IC and other autoimmune disease such as lupus erythematosis, ulcerative colitis, rheumatoid arthritis, and thyroiditis [11]. It has been shown that the urinary bladders of IC/PBS patients are infiltrated with T cells, monocytes, mast cells, and plasma cells [8], [9]. Further, the ratio of inflammatory and T helper cells to suppressor cells increases significantly in the peripheral blood of IC patients [26]. Th1 cell levels are also elevated during IC [43]. In addition, CXCR3-expressing T cells have been shown to produce predominantly Th1 cytokines and selectively mobilize Th1 and inflammatory lymphocytes [28]. IC also includes high infiltrates of T (CD4^+^ and CD8^+^) and B-cells [44]. Further, CXCR3- and Th1-dependent host responses have been observed in many models of autoimmune diseases [45], [46]. In the present study, we observed a significant increase in the number of CD4^+^ T cells at urinary bladder sites in EAC mice as compared with unaffected and/or anti-CXCL10 Ab-treated mice. However, CXCL10 blockade reduces the frequency of CD4^+^ T cells in the urinary bladder, most likely by inhibiting their chemotaxis via CXCR3 interactions. A similar effect of CXCL10 has been noted in potentiating colitis through a massive infiltration of CD4^+^ T cells, which produce Th1 cytokines [47], [48]. Also, CD4^+^ CXCR3^+^ T cells are increased in the lamina propria of IBD patients as compared with controls [49], [50]. Earlier, we have showed that increases in CD4^+^ T cells in the lamina propria of the colon during colitis [24] are mediated in part by CXCL10. Previous studies also indicate a similarity between T-cell and monocyte infiltration in IBD and IC [26], [27]. The present study showed that massive infiltration of T cells into the urinary bladder of EAC mice is significantly decreased following anti-CXCL10 Ab treatment. Taken together, the results of the present study suggest that EAC is Th1 cell/CXCR3-ligand-mediated and can be abrogated by anti-CXCL10 treatment.

Mast cells are normally distributed throughout the mucosal surface; also, connective tissues are reported to be involved in ulcer and nonulcer IC both in humans and animal models of cystitis [51]. The etiology of IC involves an increase in urinary bladder mast cell numbers [52]. Mast cells produce a variety of cytokines and chemokines that attract neutrophils [53] and T cells [54]. The mean number of mast cells increases in IC patients as compared with normal individuals [55]. Mast cells preferentially express CXCR3 protein in patients with rheumatoid arthritis and, importantly, this expression is accompanied by elevated level of CXCL9 and CXCL10 [56]. The association of mast cells with CXCR3 expression may indicate an additional mechanism for the progression of EAC. Mast cells are involved in IC of the urinary bladder [51]. Also, increases in mast cell numbers were reported in Balb/CAN mice immunized with syngeneic homogenate that cause bladder edema, fibrosis [57], and feline IC [58]. In the present study, we found a significant increase in mast cell frequency in the spleens, ILNs, and urinary bladder of EAC mice. These increases in mast cells can be abrogated by anti-CXCL10 Ab treatment. Taken together, the results of the present study clearly suggest a scenario in which EAC leads to increases in both systemic and mucosal mast cells, leading to the development of cystitis. However anti-CXCL10 Ab treatment decreases mast cell numbers in both ILNs and urinary bladders that may, in part, lead to diminished EAC severity. These effects may also be indirectly caused by the blockade of Th1-type cells within these sites, as well as the cytokines and growth factors produced in the absence of anti-CXCL10.

Neutrophils are associated with tissue injury and pathogen invasion and have been found in elevated numbers in the urine of patients with IC [59]. Recently, it has been shown that the neutrophil elastase concentration is increased in IC patients [30]. Here, we clearly show that the number of mucosal neutrophils is significantly increased after EAC induction. However, this increase declined after anti-CXCL10 Ab treatment. Our findings reinforce the hypothesis that neutrophils have the potential to regulate migration of various leukocyte types and mast cells to inflammatory sites. These effects may also be indirectly caused by the blockade of Th1 type cells within these sites, as well as by the cytokines and growth factors produced in the absence of anti-CXCL10.

In summary, the results of the present study clearly suggest that CXCL10 is partially responsible for the movement of Th1 cells (CD4^+^), mast cells, and neutrophils from the spleen and ILN to the urinary bladder, resulting in the progression of EAC severity. In particular, flow cytometry analysis showed that the percentage of CD4^+^ T cells is reduced in the ILN of EAC mice as compared to similar sites in anti-CXCL10 Ab-treated mice. In contrast, the numbers of CD4^+^ T cells, mast cells, and neutrophils were dramatically increased in the urinary bladders of EAC mice as compared with the numbers of such cells in the urinary bladders of unaffected and/or anti-CXCL10 Ab-treated mice. Overall, this study suggests that CXCL10 is a mediator of CD4^+^ T cell, mast cell, and neutrophil infiltration to the urinary bladder (effector) during cystitis. Our results also highlight the importance of CXCR3 ligand interactions in EAC, a new and potentially useful model for IC study, and present strong case for more detailed clinical study of CXCL10, which will help to develop noninvasive biomarkers of IC severity.

## Materials and Methods

### Animals and Ethics Statement

SWXJ (H-2^QS^) mice were generated by mating SJL/J (H-2^s^) males with SWR/J (H-2^q^) females at Jackson Laboratories in Bar Harbor, ME. Eight-week-old female mice were brought to the University of South Carolina School of Medicine animal facility. Animals were housed and maintained in isolator cages under normal light and dark cycles under conventional housing conditions to minimize animal pain and distress. The Institutional Animal Care committee (ICAUC) of the University of South Carolina School of Medicine approved this study. The resident veterinarian and an author monitored the pain and distress of mice each day. Experimental groups consisted of five mice each. Each study was repeated three times.

### Induction of Experimental Autoimmune Cystitis (EAC)

Bladders from 8- to 10-week-old female SWXJ (H-2^q.s^) mice were homogenized in distilled water (DW). The homogenate was centrifuged at 1,000 g for 10 min and the supernatant lyophilized overnight. The lyophilate was dissolved in double-DW so that each bladder was reduced to 100 µL. 10-week-old SWXJ 6 female mice were immunized (s.c.) in the abdominal flank with complete Freund’s adjuvant (CFA) containing 400 µg of *Mycobacterium tuberculosis* H37RA (Difco, Detroit, MI). A similar number of age- and sex-matched mice were immunized with an emulsion containing CFA alone to serve as controls. These mice have been reported to develop EAC four months after the transfer of urinary bladder homogenate [15]. Mouse behavior (pain, grooming, guarding) that could reflect symptoms of EAC was monitored twice a week until the end of experiments (data not shown). At the end of experiments, mice were euthanized by an overdose of isoflurane followed by cervical dislocation. Blood was collected to determine systemic cytokines by ELISA assays.

### CXCL9, CXCL10, and CXCL11 ELISAs

Serum concentrations of mouse CXCL9, CXCL10 and CXCL11 were determined by Enzyme-linked immunosorbent assay (ELISA) assays (R&D Systems, Minneapolis, MN) according to the manufacturer’s instructions. In brief, samples and standards were added to the wells and plates and incubated for 2 hr at room temperature (RT). Conjugate-detection solutions were added to each well and the plates were further incubated for 2 hr at RT. Substrate solutions were then added to each well and plates were incubated for 30 min. Stop solution was added to each well of and the plates were read at an optical density of 450 nm after 30 min, using a λ correction of 540 and 570 nm. ELISA assays were capable of detecting >10 pg/ml of each chemokine.

### Anti-CXCL10 Ab Treatment

We used anti-CXCL10 Ab as described elsewhere [23]. In brief, three months after the induction of EAC, mice received intraperatoneal anti-CXCL10 Ab or pre-immune sera treatment twice a week for eight weeks until the end of experiments. Behavior that could reflect severity of EAC (pain, grooming, guarding) (data not shown) was monitored twice weekly until the end of experiments.

### RNA Isolation and Gene Expression Analysis

Total RNA was isolated from spleens, ILNs, and urinary bladders of EAC. Normal mice were treated with either anti-CXCL10 or control Ab, using Tri-reagent™ (Molecular Research Center, Cincinnati, OH), according to the manufacturer’s protocol. Potential genomic DNA contamination was removed from these samples by treating them with RNase-free DNase (Invitrogen, San Diego, CA) for 15 min at 37°C. RNA was precipitated and resuspended in RNA Secure™ (Ambion, Austin, TX). cDNA was generated by reverse transcribing approximately 1.5 µg of total RNA using Taqman™ reverse transcription reagent (Applied Biosystems, Foster City, CA) according to the manufacturer’s protocol. Mouse mRNA sequences of CXCL9, CXCL10, CXCL11, CXCR3, IFN-γ, IL-12p40, TNF-α, and 18S rRNA were obtained from the NIH National Center for Biotechnology Information (NCBI) gene bank database. These sequences were used to design primers for real-time polymerase chain reaction (RT-PCR) analysis of CXCL9, CXCL10, CXCL11, TNF-α, IFN-γ, IL-12p40, CXCR3 mRNAs, and 18S rRNAs, respectively. Details of the primer and analysis methods are described elsewhere [23].

### Histological Alterations

Mouse urinary bladders were evaluated for inflammatory cell infiltrates and interstitial edema. Urinary bladders were preserved using 10% neutral formalin fixative for 24 hr and embedded in paraffin. Fixed tissues were sectioned at 6 µm, stained with hematoxylin and eosin, and examined by light microscopy. The inflammatory state of each urinary bladder was characterized and scored as follows: having no change as compared with tissue samples from untreated mice (score = 0); having a few multi-focal mononuclear cell infiltrates (score = 1); having minimal hyperplasia with a mixture of mononuclear and multi-nucleated cells (score = 2); having major hyperplasia (score = 3); or having major hyperplasia and epithelial erosions with inflammation in the submucosa (score = 4).

### Cell Isolation and Flow Cytometry

Single-cell suspensions of spleens, ILN, and urinary bladders from each mouse were passed through a sterile wire screen (Sigma, USA]). Cell suspensions were washed twice in RPMI 1640. Urinary bladder lymphocytes were further purified using a discontinuous Percoll gradient (Pharmacia, Uppsala, Sweden) and collected at the 40% to 75% interface. Lymphocytes were maintained in complete medium consisting of RPMI 1640 supplemented with 10 ml/L of nonessential amino acids (Mediatech, Washington, DC), 1 mM sodium pyruvate (Sigma, USA), 10 mM HEPES (Mediatech, Herndon, VA), 100 U/ml penicillin, 100 µg/ml streptomycin, 40 µg/ml gentamycin (Elkins-Sinn, Cherry Hill, NJ), 50 µM mercaptoethanol (Sigma), and 10% fetal bovine serum (FBS) (Sigma, USA). Cells were stained with phycoerythrin cyanine-5 (PE-Cy5)-conjugated anti-CD4 (H129.19), allophycocyanin (APC) conjugated anti-LY6G (RB6–8C5), APC-conjugated rat anti-mouse CD117, and fluorescein isothiocynate (FITC) conjugated anti-CD3 (145-2C11) for 30 min with shaking. Lymphocytes were then washed with FACS buffer (PBS with 1% BSA) and fixed in 2% paraformaldehyde (Sigma) in PBS and analyzed by flow cytometry (Becton Dickinson, San Diego, CA).

### Statistical Analysis

The traditional α-value (i.e., *p*<0.01) was used to evaluate statistical significance. Data are expressed as the mean ± SD and compared using a two-tailed paired student’s *t*-test or an unpaired Mann Whitney *U*-test. The results were analyzed using Microsoft Excel (Microsoft, Seattle, WA) for Macintosh computers. Single-factor and two-factor ANOVA analyses were used to evaluate groups and subgroups, respectively. The Kolmogorov-Smirnov (K-S) two-sample test using CXP analysis Software (Beckman Coulter) was used to compute statistical significance between histograms.
